# Synthesis and Photoluminescent Properties of Dy^3+^-Doped and Dy^3+^/Eu^3+^ Co-Doped 50ZnO:40B_2_O_3_:5WO_3_:Nb_2_O_5_ Glass

**DOI:** 10.3390/molecules30102229

**Published:** 2025-05-20

**Authors:** Margarita Milanova, Aneliya Yordanova, Lyubomir Aleksandrov, Reni Iordanova, Petia Petrova

**Affiliations:** 1 Institute of General and Inorganic Chemistry, Bulgarian Academy of Sciences, G. Bonchev Str., Bld. 11, 1113 Sofia, Bulgaria; margi@svr.igic.bas.bg (M.M.); lubomirivov@gmail.com (L.A.); reni@svr.igic.bas.bg (R.I.); 2 Institute of Optical Materials and Technologies “Acad. Jordan Malinowski”, Bulgarian Academy of Sciences, Blvd. Akad. G. Bonchev Str., Bld. 109, 1113 Sofia, Bulgaria; petia@iomt.bas.bg

**Keywords:** glass, rare earths, photoluminescence, density

## Abstract

Dy^3+^ single-doped and Dy^3+^/Eu^3+^ co-doped ZnO:B_2_O_3_:WO_3_:Nb_2_O_5_ glass was successfully synthesized using the melt quenching method. The amorphous character of the prepared samples was confirmed by X-ray diffraction (XRD). The glass transition and crystallization temperatures were examined by differential scanning calorimetry (DSC). Raman spectroscopy was applied to investigate the glass microstructure. Physical properties like the density, molar volume, oxygen molar volume and oxygen packing density of the glass were also determined. The photoluminescence excitation (PLE) and emission (PL) spectra of the resultant glass types were measured. The obtained Dy^3+^ single-doped glass was characterized by strong luminescence at 482 and 574 nm, corresponding to the ^4^F_9/2_ → ^6^H_15/2_ (blue) and ^4^F_9/2_ → ^6^H_13/2_ (yellow) transitions, respectively, and weak luminescence at 663 nm and 753 nm due to the ^4^F_9/2_ → ^6^H_11/2_ (red) and ^4^F_9/2_ → ^6^H_9/2_ + ^6^F_11/2_ (red) transitions. The luminescence results indicate that energy transfer from the Dy^3+^ to Eu^3+^ ions occurs in the proposed glass system. The emitted light from the Dy^3+^ single-doped glass was found to be yellow-orange. The Dy^3+^/Eu^3+^ co-doped samples emitted darker orange light. The obtained results show that the investigated types of glass have the potential to be used as orange light-emitting materials.

## 1. Introduction

For many years, luminescent materials doped with rare-earth (RE) elements have garnered significant attention because of their diverse applications [1,2]. These light-emitting materials are used in a variety of areas, including optoelectronic devices, displays, biomarkers, optical fibers and lasers [3,4,5,6,7]. A considerable part of the research in this domain is dedicated to rare-earth-ion-doped phosphors for solid-state lighting (SSL)-based devices, such as light-emitting diodes (LEDs) [8,9]. These materials offer several benefits compared to traditional incandescent or fluorescent lamps, including high brightness, efficiency, a long lifespan, reliability and reduced energy consumption [10]. This aspect is particularly vital considering that the electrical lighting industry accounts for nearly 19% of global electricity usage [11]. Consequently, the creation of innovative and environmentally friendly materials that are both efficient and durable is of utmost importance [2,12,13,14]. In this context, the significance of RE ions is considerable. These ions possess a distinctive capability to emit light due to their 4f-4f or 4d-4f transitions when excited at an appropriate wavelength [8,15]. Dy^3+^ single-doped materials can produce neutral white light emission. The luminescence spectrum of trivalent dysprosium ions reveals multiple characteristic lines, with two main bands appearing in the blue (470–500 nm) and yellow (570–600 nm) regions, corresponding to the transitions ^4^F_9/2_ → ^6^H1_5/2_ and ^4^F_9/2_ → ^6^H_13/2_, respectively [16,17]. White light emission can be achieved through the proper adjustment of the blue and yellow luminescence intensity ratio [15,18,19]. Nonetheless, there is often a preference for producing warm white light rather than a cold, artificial white hue [20]. To generate this warm light, red emitters such as Eu^3+^ ions can be utilized, as their strongest band related to the ^5^D_0_ → ^7^F_2_ transition of Eu^3+^ occurs at approximately 615 nm, placing it within the red section of the visible light spectrum [21,22]. Furthermore, a suitable ratio of Eu^3+^/Dy^3+^ within the host matrix can facilitate color adjustment, resulting in the emission of warm white light. This phenomenon has been observed in various Dy^3+^/Eu^3+^-doped matrices, such as yttrium alumino bismuth borosilicate and CdO-GeO_2_-TeO_2_ glass [4,23], as well as phosphate glass [24]. Additionally, warm white light was achieved in lithium fluoride bismuth borate glass co-doped with Dy^3+^ and Eu^3+^ [25]. Research on Zn(PO_3_)_2_ glass doped with Eu^3+^ and Dy^3+^ ions shows the transition of white light emission from neutral white at 348 nm excitation to warm white at 445 nm [26]. On the other hand, when a pair of rare-earth (RE) ions have matched energy levels and are co-doped in a host, a process in which one RE is sensitized by the energy from the other ion may occur. This process is defined as energy transfer (ET), a common way to improve the luminescence properties of RE ion-doped materials [27]. Combining Dy^3+^ with Eu^3+^ tends to enforce the europium emission because when materials with Dy^3+^ and Eu^3+^ are excited with a UV light source, the Dy^3+^ ions absorb the incident light, and they non-radiatively transfer part of the absorbed energy to Eu^3+^ ions, causing emissions.

It is well known that binary ZnO-B_2_O_3_ glass has been attracting continuous scientific interest as a host for luminescence applications due to its high transparency from the visible to mid-infrared region of the spectrum, its relatively low melting temperature, and its good chemical and thermal stability. We have experience in the synthesis, structural analysis and luminescent properties of ZnO-B_2_O_3_ glass containing WO_3_ and Nb_2_O_5_ doped with Eu^3+^ ions [28]. Our results have shown the existence of a charge transfer from the matrix (WO_3_ and Nb_2_O_5_) to Eu^3+^, resulting in enhanced Eu^3+^ emission. We are also expecting the occurrence of such a charge transfer from the matrix to Dy^3+^ (which we established through spectral results). On the other hand, it is well known from the literature that in a Dy^3+^ and Eu^3+^ co-doped matrix, a non-radiative charge transfer from Dy^3+^ to Eu^3+^ active ions is observed, resulting in an increase in Eu^3+^ emission intensity. In this way, we are sensitizing Eu^3+^ in two ways—from the matrix and from Dy^3+^. The novelty and significance of this study is the composition of the matrix, which is suitable for hosting Eu^3+^ and Dy^3+^ active ions and can also serve as a host sensitizer of both ions. The aim of this work was to synthesize ZnO:B_2_O_3_:WO_3_:Nb_2_O_5_ glass doped with different concentrations of Dy^3+^ ions and co-doped with Eu^3+^ ions, which could be used as a phosphor in LEDs. This study provides a detailed analysis of the thermal and luminescent properties and structural features of these prepared types of glass. Physical parameters such as the density, molar volume, oxygen molar volume and oxygen packing density, which provide some structural information, were also evaluated.

## 2. Results

### 2.1. XRD Data and Thermal Analysis

Two series of glass were obtained. Their compositions and respective names are listed in Table 1 and Table 2, respectively.

**Figure 1:**
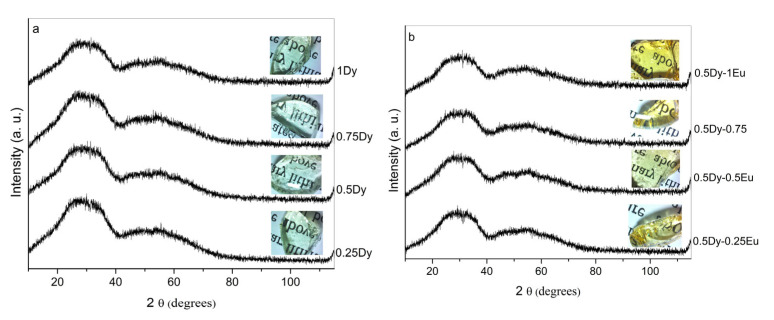
XRD patterns of the investigated glasses: (**a**) Dy^3+^-doped 50ZnO:40B_2_O_3_:5WO_3_:5Nb_2_O_5_ glass; (**b**) Dy^3+^/Eu^3+^ co-doped 50ZnO:40B_2_O_3_:5WO_3_:5Nb_2_O_5_ glass.

The measured X-ray diffraction patterns are shown in Figure 1a,b. The absence of sharp diffraction peaks in the spectra showed that prepared samples are glassy in nature.

**Figure 2:**
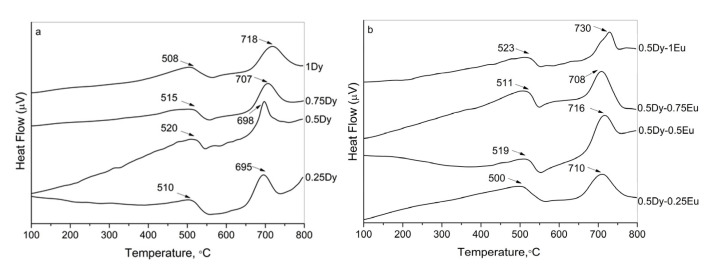
DSC curves of the investigated glasses: (**a**) Dy^3+^-doped 50ZnO:40B_2_O_3_:5WO_3_:5Nb_2_O_5_ glass; (**b**) Dy^3+^/Eu^3+^ co-doped 50ZnO:40B_2_O_3_:5WO_3_:5Nb_2_O_5_ glass.

The amorphous nature of the obtained materials was further confirmed by differential scanning calorimetry (DSC). The DSC curves for the two series of samples—Dy^3+^-doped and Dy^3+^/Eu^3+^ co-doped glasses—are shown in Figure 2a and Figure 2b, respectively.

The endothermic dips corresponding to the glass transition temperature (T_g_) and the exothermic peaks due to the crystallization temperature (T_c_) are observed. The estimated values of T_g_ and T_c_ are pointed out in the figure. As can be seen from the figure, the glass transition temperatures of all glasses are above 500 °C and do not vary significantly with composition, indicating that the addition of Dy_2_O_3_ and Eu_2_O_3_ does not significantly affect the structure of the matrix glass. All glasses are characterized by a high thermal stability, i.e., ΔT = T_c_ − T_g_ between 178 and 210 °C.

### 2.2. Physical Properties

Structural information of the glasses was gained by density (*ρ_g_*) measurements, which provided the base for the determination of several physical parameters, such as molar volume (*V_m_*), oxygen molar volume (*V_o_*) and oxygen packing density (*OPD*). These were evaluated using the following relations:

(1) $$V_{m}=\frac{\sum x_{i}M_{i}}{\rho_{g}}$$

(2) $$V_{o}=V_{m}\times \left(\frac{1}{\sum x_{i}n_{i}}\;\right)$$

(3) $$OPD=1000\times C\times \left(\frac{\rho_{g}}{M}\right)$$

where *x_i_* is the molar fraction of each component *i*, *M_i_* is the molecular weight, *ρ_g_* is the glass density, *n_i_* is the number of oxygen atoms in each oxide, *C* is the number of oxygen atoms per formula units, and *M* is the total molecular weight of the glass compositions. The values obtained are listed in Table 3 and Table 4.

As seen from Table 3, the density of glass 50ZnO:40B_2_O_3_:5WO_3_:5Nb_2_O_5_ from the first series with increasing content of Dy_2_O_3_ increases with the Dy_2_O_3_ loading, which is mostly due to the increase in average molecular mass of the glasses [29]. Molar volume (*V_m_*) is also an important physical property and normally follows a trend opposite to that of density. However, *V_m_* also enhances with increasing Dy_2_O_3_ content, indicating an increasing inter-atomic spacing in the network due to the insertion of Dy^3+^ ions with high ionic radius (0.91 Å). Oxygen molar volume (*V_o_*) and *OPD* are two parameters that provide information on the packing of the oxygen ions in the glass structure [30]. Lower *V_o_* and higher OPD indicate a higher degree of network connectivity. In this series of glasses, the lowest *V_o_* and the highest *OPD* are observed for the Dy0.5 glass, evidencing the highest cross-linking and bonding in its glass network. The highest value of *V_o_* and the lowest *OPD* for Dy0.25, Dy0.75, Dy1 glasses indicate lower cross-linking density and the formation of less reticulated glass networks. For the second series of 50ZnO:40B_2_O_3_:5WO_3_:5Nb_2_O_5_ glasses, containing a constant Dy_2_O_3_ content of 0.5 mol% and increasing Eu_2_O_3_ concentration from 0.25 to 1 mol% (Table 4), both density and oxygen molar values increase with the Eu_2_O_3_ loading. *V_o_* increases and *OPD* decreases for glasses having up to 0.75 mol% Eu_2_O_3_ (samples 0.5Dy-0.25Eu_2_O_3_, 0.5Dy-0.5Eu_2_O_3_ and 0.5Dy-0.75Eu_2_O_3_). With future increase in Eu_2_O_3_ content of 1 mol% (sample 0.5Dy-1Eu), the oxygen molar volume decreases and oxygen packing density enhances. The increasing glass density can be attributed to the increase in the average molecular mass of glasses [29]. As the molar volume (volume occupied by a mass of the glass equal to 1 mole) is strongly affected by the ionic radii of the incorporated ionic species in the glass, the increasing trend of *V_m_* is due to the insertion of Eu^3+^ ions, which are known to have a high ionic radius (0.95 Å), resulting in the formation of an excess free volume, which increases the overall molar volume of these glasses [31]. The increasing *V_o_* and decreasing *OPD* for glasses having up to 0.75 mol% Eu_2_O_3_ revealed insufficient cross-linking (high number of non-bridging oxygen atoms) of the borate network. Decreasing *V_o_* and enhancing *OPD* for Dy0.5-1Eu glass suggest increased cross-linking in the glass network.

### 2.3. Raman Analysis

**Figure 3:**
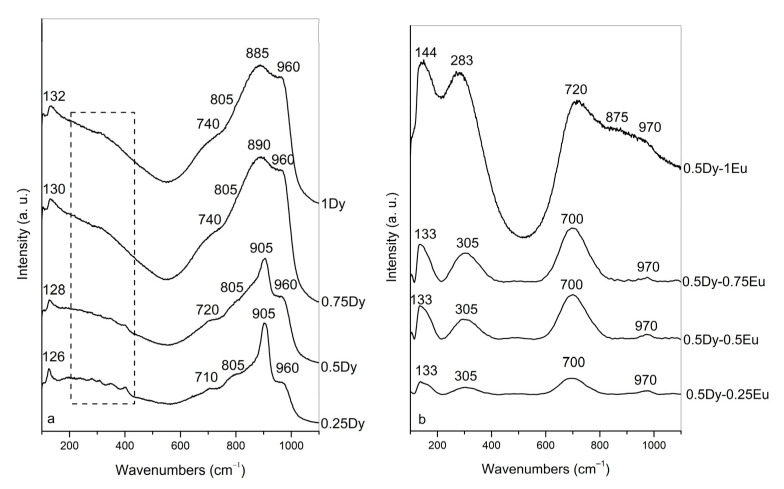
Raman spectra of the investigated glasses: (**a**) Dy^3+^-doped 50ZnO:40B_2_O_3_:5WO_3_:5Nb_2_O_5_ glass; (**b**) Dy^3+^/Eu^3+^ co-doped 50ZnO:40B_2_O_3_:5WO_3_:5Nb_2_O_5_ glass.

The structure of the obtained glasses was studied by Raman spectroscopy. Figure 3a shows the Raman spectra of 50ZnO:40B_2_O_3_:5WO_3_:5Nb_2_O_5_ glasses from the first series, containing increasing Dy_2_O_3_ content. As can be seen, all glass spectra contain a band at 960 cm^−1^, a high-intensity band at 905–885 cm^−1^, a shoulder at 805 cm^−1^, a broad shoulder at 710–740 cm^−1^, broad Raman activity in the range of 200–450 cm^−1^ and a band at 126–132 cm^−1^. Taking into account previous spectral investigations on glasses 50ZnO:40B_2_O_3_:5WO_3_:5Nb_2_O_5_:xEu_2_O_3_ (x = 0, 0.1, 0.5, 1, 2, 5, and 10 mol%) and (50-x)WO_3_:25La_2_O_3_:25B_2_O_3_:xNb_2_O_5_ reported in refs. [28,32], we attribute the band at 960 cm^−1^ in the Raman spectra of the first series glasses to the ν_1_ mode of the isolated tetrahedral species, [WO_4_]^2−^. This band becomes more intense with the addition of Dy_2_O_3_, indicating an increasing quantity of isolated [WO_4_]^2−^ groups as a result of the WO_6_ → WO_4_ transformation. The intensive Raman peak at 905–885 cm^−1^ is assigned to overlapping contributions from the asymmetric stretching mode ν_3_ of [WO_4_]^2−^ tetrahedra and the ν_1_ stretching vibration of short Nb–O bonds in strongly deformed and isolated NbO_6_ octahedra [28,32]. The broadening of this band with increasing Dy_2_O_3_ content indicates the formation of [WO_4_]^2−^ tetrahedral and NbO_6_ octahedral units with different distortions upon the addition of Dy_2_O_3_. The shoulder at 805 cm^−1^ is a superposition of the symmetric stretching ν_1_ mode of tetrahedral [NbO_4_]^3−^ groups, and vibrations of B–O–Nb bonds [28,32]. The broad Raman shoulder at ca. 710–740 cm^−1^ is attributed to the stretching of the bridging oxygen atoms linking the tungsten ion and niobium polyhedral units, via Nb–O–W bridges that overlap with the vibrations of chain-type metaborate units, [BØ_2_O]^−^ [28,33]. In addition, the observed upshift to 740 cm^−1^ indicates that metaborate units, [BØ_2_O]^−^, are charge balanced by Dy^3+^ ions. The increasing intensity of this shoulder upon increasing Dy_2_O_3_ concentration indicates a growing number of Nb–O–W linkages and metaborate groups in the glass networks. The vibrational features in the lower-frequency region 200–450 cm^−1^ correspond mainly to bending modes of [WO_4_]^2−^ and [NbO_4_]^3−^ tetrahedra coupled with the Zn–O (at ca. 235 cm^−1^) and Dy–O vibrations (378 cm^−1^) [28,34,35]. The band observed at 126–132 cm^−1^ in all glass compositions was related to the out-of-plane W–O deformation mode [36].

Figure 3b presents the Raman spectra of the second series of 50ZnO:40B_2_O_3_:5WO_3_:5Nb_2_O_5_ glasses containing a constant Dy_2_O_3_ content of 0.5 mol% and increasing Eu_2_O_3_ concentration from 0.25 to 1 mol%. As can be seen from the figure, all glass spectra contain Raman bands at 133–144 cm^−1^, at 305–283 cm^−1^, at 700–720 cm^−1^ and at 970 cm^−1^. In the spectrum of glass 0.5Dy-1Eu, a band is also present at about 875 cm^−1^ that is not well resolved.

Having in mind our previous spectral investigation on glasses 50ZnO:40B_2_O_3_:5WO_3_:5Nb_2_O_5_:xEu_2_O_3_ (x = 0, 0.1, 0.5, 1, 2, 5, and 10 mol%) reported in ref. [28], we ascribe the weak band at 970 cm^−1^ to the ν_1_ mode of the isolated tetrahedra, [WO_4_]^2−^. The not-well-resolved band at 875 cm^−1^ is due to a coupled mode including the ν_1_ stretching vibration of short Nb–O bonds in strongly deformed and isolated NbO_6_ octahedra and the asymmetric stretching mode ν_3_ of [WO_4_]^2−^ tetrahedra [28]. The band at 700–720 cm^−1^ is a superposition of the peaks associated with the vibrational modes of the Nb–O–W linkages and metaborate units [28,33]. The band at 305–283 cm^−1^ is connected with the overlapping Zn–O, Dy–O, and Eu–O stretching vibrations [28,35,37]. The low- frequency band at 133–144 cm^−1^ is assigned to the out-of-plane W–O deformation mode [36]. As seen from Figure 3b, with increasing Eu_2_O_3_ content, the overall intensity of the Raman spectra for all glasses increases, reflecting enhanced crosslinking in the glass network [38]. In addition, the observed upshift of the band at 700 to 720 (vibrations of Nb–O–W and [BØ_2_O]^−^) and the band at 133 to 144 (W–O deformation mode) in the Raman spectrum of the glass having the highest Eu_2_O_3_ content of 1 mol% (Figure 3b, spectrum 0.5Dy-1Eu_2_O_3_) indicates that the short-range order of niobate, tungstate and borate groups is influenced by the added Eu_2_O_3_. Most probably, the Eu^3+^ ions are situated in the local environment of these structural arrangements, charge balancing them via the formation of Nb/W–O–Eu and B–O–Eu bonding and thus increasing the reticulation of the glass network. This observation coincides well with the physical parameters obtained above and the data of the thermal analysis.

### 2.4. Photoluminescent Properties

**Figure 4:**
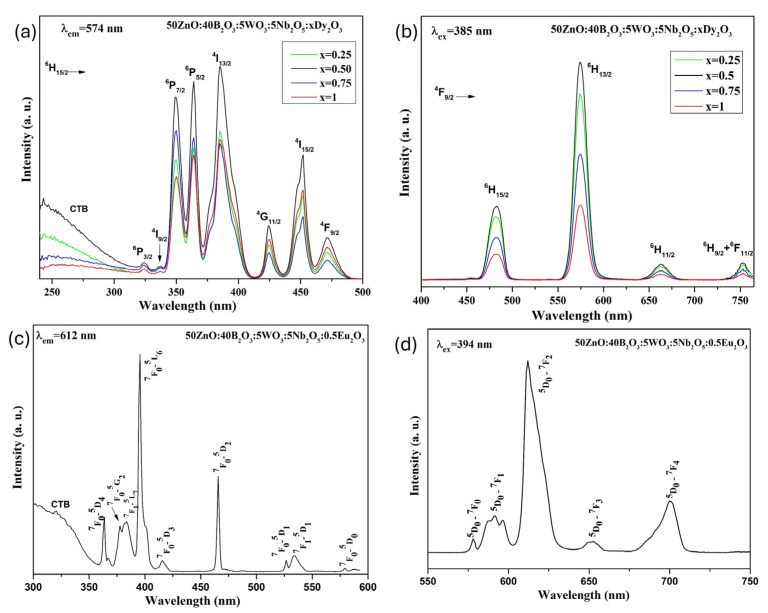
(**a**) Excitation spectra of Dy^3+^-doped 50ZnO:40B_2_O_3_:5WO_3_:5Nb_2_O_5_ glass; (**b**) emission spectra of Dy^3+^-doped 50ZnO:40B_2_O_3_:5WO_3_:5Nb_2_O_5_ glass; (**c**) excitation spectrum of Eu^3+^-doped 50ZnO:40B_2_O_3_:5WO_3_:5Nb_2_O_5_ glass; (**d**) emission spectrum of Eu^3+^-doped 50ZnO:40B_2_O_3_:5WO_3_:5Nb_2_O_5_ glass.

The excitation spectra of Dy^3+^-doped 50ZnO:40B_2_O_3_:5WO_3_:5Nb_2_O_5_ glass, monitored at the 574 nm wavelength, are shown at Figure 4a. The sharp excitation peaks seen at 324 nm, 337 nm, 349 nm, 363 nm, 385 nm, 424 nm, 452 nm and 471 nm can be attributed to the transitions of ^6^H_15/2_ → ^6^P_3/2_, ^6^H_15/2_ → ^4^I_9/2_, ^6^H_15/2_ → ^4^P_7/2_, ^6^H_15/2_ → ^6^P_5/2_, ^6^H_15/2_ → ^4^I_13/2_, ^6^H_15/2_ → ^4^G_11/2_, ^6^H_15/2_ → ^4^I_15/2_, ^6^H_15/2_ → ^4^F_9/2_, respectively [39]. From the observed spectra, the intense band at 385 nm (^6^H_15/2_ → ^4^I_13/2_) was selected for photoluminescence measurements, as it shows the highest intensity.

These wavelengths are appropriate for excitation using blue LED chips (430–470 nm) and commercial near-ultraviolet light-emitting diodes (LEDs) (250–400 nm). In addition, it can be seen that the excitation spectra show a broad band below 320 nm, which corresponds to the ligand-to-metal charge transfer states (LMCT) of O^2−^ → Dy^3+^ from the oxygen 2p excited state to the Dy^3+^ 4f state [40], and O^2−^ → W^6+^ in WO_4_ groups [41] and O^2−^ → Nb^5+^ in NbO_n_ groups (NbO_n_ = NbO_6_, NbO_4_) [42]. The overlapping of bands in this spectral region makes it impossible to distinguish the individual contribution of these transitions. The existence of the host lattice absorption in the excitation spectra indicates the presence of energy transfer from the WO_4_ and NbO_n_ structural polyhedra to the active Dy^3+^ ions. This mechanism is known as host-sensitized luminescence [41,43]. Figure 4b shows the visible emission spectra of the Dy^3+^-doped 50ZnO:40B_2_O_3_:5WO_3_:5Nb_2_O_5_ glass under excitation at a wavelength of 385 nm. The obtained glasses are characterized by strong luminescence at 482 and 574 nm corresponding to the ^4^F_9/2_ → ^6^H_15/2_ (blue) and ^4^F_9/2_ → ^6^H_13/2_ (yellow) transitions, respectively, and weaker luminescence at 663 nm and 753 nm due to the ^4^F_9/2_ → ^6^H_11/2_ (red) and ^4^F_9/2_ → ^6^H_9/2_ + ^6^F_11/2_ (red) transitions. From Figure 4b it is clear that the emission intensity strongly depends on the Dy^3+^ concentration and increases with increasing Dy_2_O_3_ content, reaching a maximum at 0.5 mol%, after which it gradually decreases. This fact may be attributed to the concentration quenching effect [44]. As the Dy^3+^ concentration rises, the distance between Dy^3+^ reduces, enabling non-radiative energy transfer between neighboring Dy^3+^ ions. The occurrence of crossing-relaxation phenomena between these ions reduces the jump from the ^4^F_9/2_ energy level to ^6^H_J_ levels (J = 15/2, 13/2, 11/2 and 9/2) and ^6^F_11/2_, leading to fluorescence quenching and weakening of the emission intensity.

To study the local symmetry around rare earth ions, dysprosium can be used as a spectroscopic probe. Among all the observed emission transitions, the blue emission transition (^4^F_9/2_ → ^6^H_15/2_) at 482 nm is an allowed magnetic dipole (MD) in nature and is independent of a local crystal field environment, while the yellow emission transition at 574 nm (^4^F_9/2_ → ^6^H_13/2_) is identified as an electric dipole (ED) and is forced by the crystal field environment in the vicinity of the Dy^3+^ ions. When the yellow emission is dominant in the spectrum, the Dy^3+^ ions are located at low-symmetry sites. Conversely, when the blue emission is stronger in the PL spectrum, the Dy^3+^ ions are located at high-symmetry sites (with inversion symmetry center) [45,46]. In our case, Dy^3+^ ions are located in the more asymmetrical environment, as evidenced by the greater intensity of the ^4^F_9/2_ → ^6^H_13/2_ transition compared to the ^4^F_9/2_ → ^6^H_15/2_ emission transition.

In our previous article, the same glass composition (50ZnO:40B_2_O_3_:5WO_3_:5Nb_2_O_5_) with different Eu^3+^ doping concentrations (0–10 mol%) was obtained [28]. As an example, in Figure 4c,d can be seen the excitation and emission spectra of 0.5 Eu^3+^-doped glass. We established an increase in europium emission intensity due to the appearance of strong host lattice absorption, resulting in non-radiative energy transfer from the WO_4_ and NbO_n_ structural polyhedra to the active Eu^3+^ ion. This fact, along with the present results concerning Dy^3+^-doped glass (Figure 4a,b), shows that 50ZnO:40B_2_O_3_:5WO_3_:5Nb_2_O_5_ glass matrix is suitable for hosting both active ions and can serve as a host sensitizer. Therefore, inspired by the above-mentioned observations, in the present study, we are synthesizing Dy^3+^/Eu^3+^ co-doped glass with different active ion concentrations, which will be excited simultaneously to verify the resulting color of emission.

**Figure 5:**
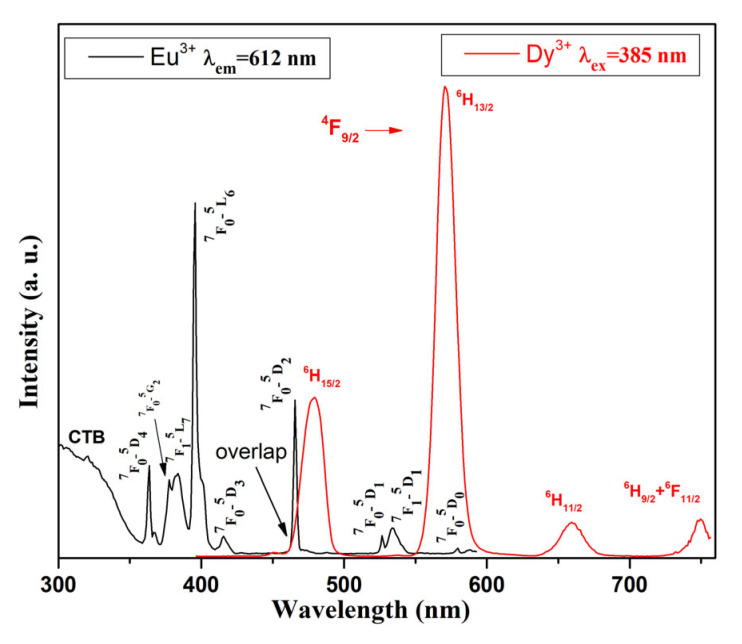
Overlap between excitation spectra of Eu^3+^-doped 50ZnO:40B_2_O_3_:5WO_3_:5Nb_2_O_5_ glass (black line) and emission spectra of Dy^3+^-doped 50ZnO:40B_2_O_3_:5WO_3_:5Nb_2_O_5_ glass (red line).

Furthermore, Figure 5 shows the overlap between the excitation spectra of Eu^3+^-doped glass (black line), monitored at 612 nm wavelength and emission spectra of Dy^3+^-doped glass (red line) at 385 nm excitation. Within the wavelength range of 450–500 nm, a spectral overlap is observed, which indicates that Eu^3+^ can be sensitized by Dy^3+^ in the obtained Dy^3+^/Eu^3+^ co-doped 50ZnO:40B_2_O_3_:5WO_3_:5Nb_2_O_5_ luminescent glass [47].

**Figure 6:**
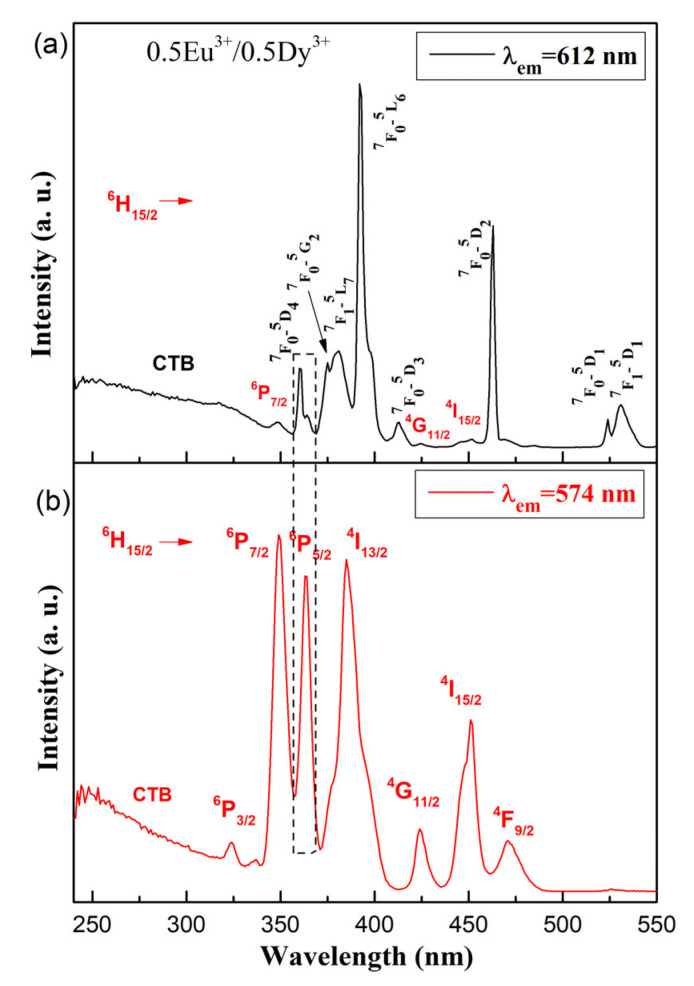
Excitation spectra of the 0.5Dy^3+^/0.5Eu^3+^ co-doped 50ZnO:40B_2_O_3_:5WO_3_:5Nb_2_O_5_ glass under (**a**) 612 nm (Eu^3+^) and (**b**) 575 (Dy^3+^) excitations.

Figure 6 shows the excitation spectra of the 0.5Dy^3+^/0.5Eu^3+^ co-doped 50ZnO:40B_2_O_3_:5WO_3_:5Nb_2_O_5_ glass at 612 nm (a) and at 574 nm (b) excitation wavelengths. When monitoring the most intensive transition for Dy^3+^ (^4^F_9/2_ → ^6^H_13/2_) at 574 nm, the excitation spectra of 0.5Dy^3+^/0.5Eu^3+^ co-doped glass consists of only Dy^3+^ bands (Figure 6b). In contrast, when monitoring the most prominent emission of Eu^3+^ (^5^D_0_ → ^7^F_2_) at 612 nm, the excitation spectrum consists of all Eu^3+^ transitions along with several low-intensity Dy^3+^ bands: ^6^H_15/2_ → ^6^P_7/2_ (349 nm), ^6^H_15/2_ → ^4^G_11/2_ (424 nm), and ^6^H_15/2_ → ^4^I_15/2_ (452 nm). The appearance of excitation wavelengths of Dy^3+^, when monitoring the Eu^3+^ emission at 612 nm, provides evidence that energy transfer from Dy^3+^ to Eu^3+^ occurs. As can be seen from Figure 6, for both Dy^3+^ and Eu^3+^, excitation peaks are located at around 363 nm.

Hence, in order to achieve better co-emission from Dy^3+^ and Eu^3+^ in the co-doped glass, the 363 nm wavelength was chosen as the excitation source.

**Figure 7:**
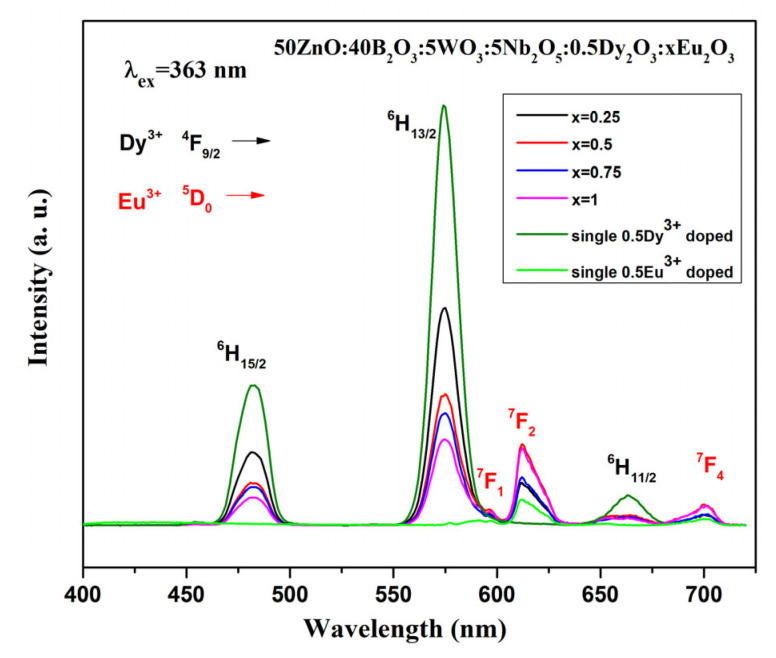
Emission spectra of 50ZnO:40B_2_O_3_:5WO_3_:5Nb_2_O_5_:0.5Dy_2_O_3_:xEu_2_O_3_ (x = 0, 0.25, 0.5, 0.75 and 1 mol%) and single Eu^3+^-doped glasses.

As can be seen from Figure 4a, the maximum emission intensity is observed at a Dy^3+^ concentration of 0.5 mol%. Based on this observation, we synthesized co-doped glass with 0.5 mol% Dy^3+^ and varying Eu^3+^ concentrations ranging from 0.25 to 1.0 mol%. The emission spectra of 0.5Dy^3+^/xEu^3+^ (x = 0, 0.25, 0.5, 0.75, and 1.0 mol%) co-doped glass and single Eu^3+^-doped glass under 363 nm excitation wavelength are shown in Figure 7. The characteristic peaks located at 482, 574 and 663 nm belong to the Dy^3+^ transitions ^4^F_9/2_ → ^6^H_15/2_, ^4^F_9/2_ → ^6^H_13/2_ and ^4^F_9/2_ → ^6^H_11/2_, respectively. In addition to the dysprosium, the ^5^D_0_ → ^7^F_1_, 5D_0_ → ^7^F_2_ and ^5^D_0_ → 7F_4_ emission peaks of europium are also present at 597 nm, 612 nm and 700 nm.

The intensity of europium emission peaks in the co-doped samples is most prominent at a concentration of 0.5 mol% Eu^3+^ ion (Figure 7). No further increase in intensity is observed at 1.0 mol%. The intensity of the Dy^3+^ transitions decreases progressively with increasing Eu^3+^ concentration, while the intensity of Eu^3+^ emission bands increases. This observation can be attributed to the fact that Dy^3+^ sensitizes Eu^3+^ and thus enhances the emission intensity of Eu^3+^. Additional evidence for the occurring energy transfer is that the single Eu^3+^-doped glass is characterized by lower emission intensity.

**Figure 8:**
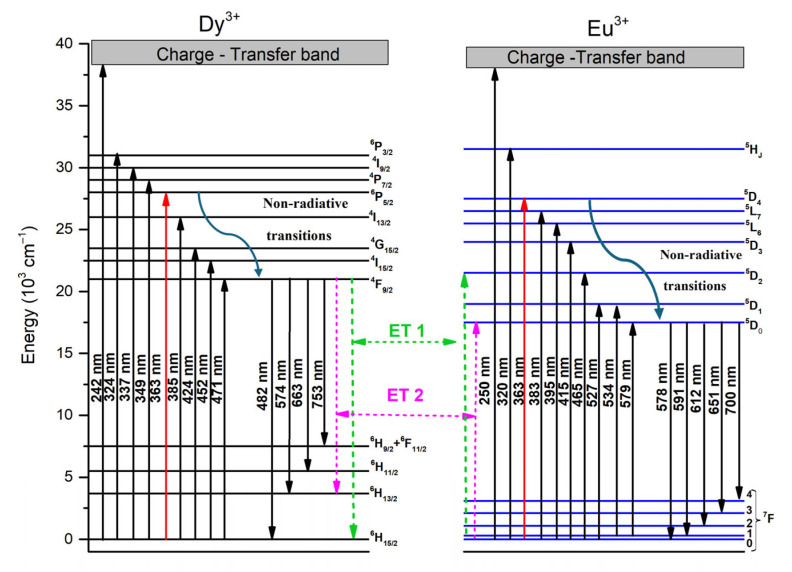
Energy transfer diagram of Dy^3+^ and Eu^3+^ co-doped glass.

Figure 8 shows the energy level diagram of Dy^3+^ and Eu^3+^ ions within the obtained luminescent glass. Under 363 wavelength of excitation, the ground state of Dy^3+^ ions was excited to the ^6^P_5/2_ excitation state and subsequently decays non-radiatively to the ^4^F_9/2_ state. From there, they emit radiatively to the 6H_J_ levels (J = 15/2, 13/2, 11/2 and 9/2) and ^6^F_11/2_. A portion of the excitation energy of the ^4^F_9/2_ state is absorbed by Eu^3+^ ions and is promoted to the ^5^D_2_ level, and after that, via non-radiative transitions, they populate the lower ^5^D_1,0_ levels and further decay radiatively to the ^7^F_J_ (J = 0, 1, 2, 3) ground states.

The energy transfer mechanisms that can occur are illustrated in Figure 8 and are as follows [48]:

ET1: ^4^F_9/2_ [Dy^3+^] + ^7^F_1_ [Eu^3+^] → ^6^H_15/2_ [Dy^3+^] + ^5^D_2_ [Eu^3+^]

ET2: ^4^F_9/2_ [Dy^3+^]+^7^F_0_ [Eu^3+^] → ^6^H_13/2_ [Dy^3+^] + ^5^D_0_ [Eu^3+^]

**Figure 9:**
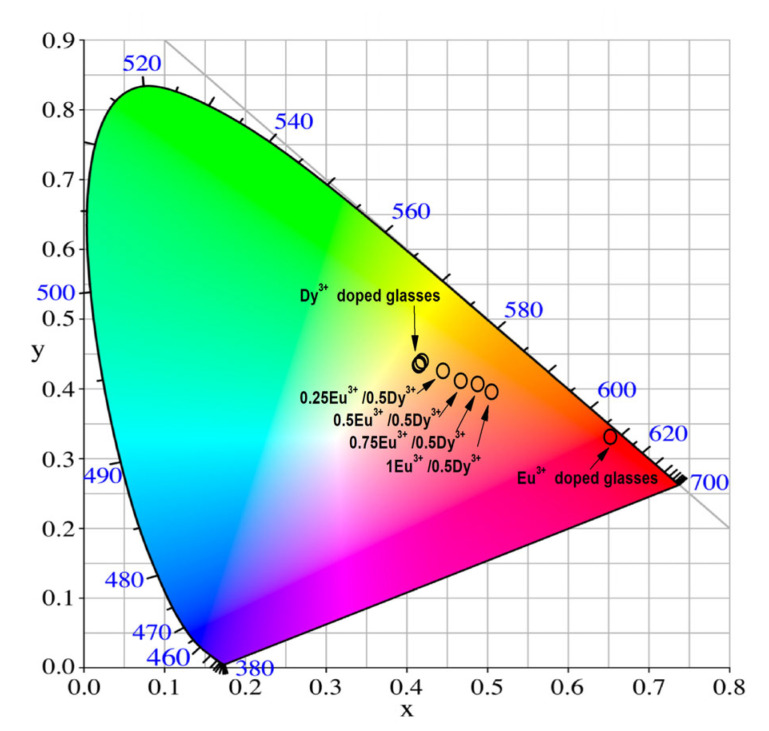
CIE chromaticity coordinates of Dy^3+^-doped glass, Eu^3+^-doped glass and Dy^3+^/Eu^3+^ co-doped 50ZnO:40B_2_O_3_:5WO_3_:5Nb_2_O_5_ glass.

To estimate the actual emission colors, the Commission International de l’Eclairage (CIE) 1931 chromaticity diagram was applied (Figure 9) [49]. The color calculator program SpectraChroma (Version 1.0.1) was employed to determine the chromaticity coordinates from the emission spectra (CIE coordinate calculator) [50]. Table 5 displays the calculated values. The emission color of the single Dy^3+^ (0.25–1 mol%)-doped glass is yellow-orange. Their values are almost identical and appear indistinguishable in the figure. When the resulting 0.5 mol% Dy^3+^-doped glass is co-doped with Eu^3+^ at a concentration of 0.25–1 mol%, the emission color becomes darker orange, and for a single Eu^3+^-doped glass, the color is pure red. The obtained results show that the investigated glass has the potential to be used as orange-emitting materials, with tunable color output, depending on the rare earth doping proportion.

## 3. Materials and Methods

Glasses with nominal composition 50ZnO:40B_2_O_3_:5WO_3_:5Nb_2_O_5_:xDy_2_O_3_ (x = 0.25, 0.5, 0.75, 1 mol%) and 50ZnO:40B_2_O_3_:5WO_3_:5Nb_2_O_5_:0.5Dy_2_O_3_:xEu_2_O_3_ (x = 0.25, 0.5, 0.75, 1 mol%) were obtained by the conventional melt-quenching method, using commercial powders of reagent grade WO_3_, ZnO, H_3_BO_3_, Nb_2_O_5_, Dy_2_O_3_ and Eu_2_O_3_ as starting materials. The homogenized batches were melted at 1250 °C for 30 min in a platinum crucible in air. The melts were cast into a graphite mold to produce bulk glass samples. Then, the glasses were transferred to a laboratory electric furnace, annealed at 490 °C (a temperature 10 °C below the glass transition temperature), and cooled to room temperature at a very slow cooling rate of about 0.5 °C/min. The phase of the samples was established by X-ray phase analysis with a Bruker D8 Advance diffractometer (Karlsruhe, Germany) using Cu Kα radiation in the 10° < 2θ < 80° range. The differential scanning calorimetry (DSC) was performed using NETZSCH Jupiter STA 449 F5 apparatus (Selb, Germany) with a Pt/Pt-Rh thermocouple in a platinum crucible with a cover. The sample was heated from room temperature up to 800 °C at a heating rate of 10 K/min under an atmosphere of argon. The density of the obtained glasses at room temperature was measured by Archimedes’ principle using toluene (ρ = 0·867 g/cm^3^) as an immersion liquid on a Mettler Toledo electronic balance (Parramatta, NSW, USA) of sensitivity 10^−4^ g. The Raman spectra were recorded on a Via Qontor Raman Confocal Microscope (Renishaw plc, Wotton-under-Edge, UK) with a laser wavelength of 532 nm (Nd:YAG-Laser, London, UK). Photoluminescence (PL) excitation and emission spectra at room temperature were measured for all glasses with a Spectrofluorometer FluoroLog3-22, Horiba, Jobin Yvon (Longjumeau, France). 

## 4. Conclusions

In summary, 50ZnO:40B_2_O_3_:5WO_3_:5Nb_2_O_5_ luminescent glasses containing Dy^3+^ and Dy^3+^/Eu^3+^ were prepared by the standard melt-quenching method. The densities are in the range of 3.887–3.991 g/cm^3^. The glass transition temperature for all glasses is over 500 °C, while the crystallization temperature, T_c_, varies between 695 and 730°C. All glasses are characterized by a high thermal stability, i.e., ΔT = T_c_ − T_g_ between 178 and 210 °C. Raman analysis revealed that the glass network consists of [WO_4_]^2−^ and [NbO_4_]^3−^ tetrahedral units, NbO_6_ octahedra, and metaborate units, [BØ_2_O]^−^. The existence of Dy^3+^ → Eu^3+^ energy transfer was confirmed by the fact that in the co-doped glasses, intensity of the Dy^3+^ transitions decreases progressively with increasing Eu^3+^ concentration, while the intensity of Eu^3+^ emission bands increases. Depending on the rare earth doping content, the researched glasses may be used as orange light-emitting materials. The CIE coordinates may be modified by altering the dopant ratio and the obtained color shifted from yellow-orange to the dark orange light region.

## Figures and Tables

**Table 1:** Molar compositions of the investigated Dy^3+^ single-doped 50ZnO:40B_2_O_3_:5WO_3_:5Nb_2_O_5_ glass (mol%).

Sample ID	ZnO	B_2_O_3_	WO_3_	Nb_2_O_5_	Dy_2_O_3_	Eu_2_O_3_
0.25Dy	50	40	5	5	0.25	-
0.5Dy	50	40	5	5	0.5	-
0.75Dy	50	40	5	5	0.75	-
1Dy	50	40	5	5	1	-

**Table 2:** Molar compositions of the investigated Dy^3+^/Eu^3+^ co-doped 50ZnO:40B_2_O_3_:WO_3_:Nb_2_O_5_ glass (mol%).

Sample ID	ZnO	B_2_O_3_	WO_3_	Nb_2_O_5_	Dy_2_O_3_	Eu_2_O_3_
0.5Dy-0.25Eu	50	40	5	5	0.5	0.25
0.5Dy-0.5Eu	50	40	5	5	0.5	0.5
0.5Dy-0.75Eu	50	40	5	5	0.5	0.75
0.5Dy-1Eu	50	40	5	5	0.5	1

**Table 3:** Values of physical parameters of 50ZnO:40B_2_O_3_:5WO_3_:5Nb_2_O_5:_xDy_2_O_3_ (0 ≤ x ≤ 1) glass: density, *ρ_g_*, molar volume, *V_m_*, oxygen molar volume, *V_o_*, oxygen packing density, *OPD*.

Sample ID	*ρ_g_*	*V_m_*	*V_o_*	*OPD*
0.25Dy	3.887 ± 0.005	24.28	11.51	86.90
0.5Dy	3.923 ± 0.004	24.29	11.46	87.28
0.75Dy	3.926 ± 0.004	24.51	11.56	86.50
1Dy	3.949 ± 0.003	24.60	11.55	86.59

**Table 4:** Values of physical parameters of 50ZnO:40B_2_O_3_:5WO_3_:5Nb_2_O_5_:0.5Dy_2_O_3_:xEu_2_O_3_ (0 ≤ x ≤ 1) glass: density, *ρ_g_*, molar volume, *V_m_*, oxygen molar volume, *V_o_*, oxygen packing density, *OPD*.

Sample ID	*ρ_g_*	*V_m_*	*V_o_*	*OPD*
0.5Dy-0.25Eu	3.927 ± 0.004	24.49	11.46	87.28
0.5Dy-0.5Eu	3.956 ± 0.002	24.53	11.52	86.83
0.5Dy-0.75Eu	3.968 ± 0.003	24.68	11.53	86.71
0.5Dy-1Eu	3.991 ± 0.003	24.76	11.52	86.83

**Table 5:** CIE chromaticity coordinates of Dy^3+^ and Eu^3+^ single-doped and Dy^3+^/Eu^3+^ co-doped 50ZnO:40B_2_O_3_:5WO_3_:5Nb_2_O_5_ glass.

Glass Composition	λ_ex_ (nm)	Chromaticity Coordinates (x, y)
50ZnO:40B_2_O_3_:5WO_3_:5Nb_2_O_5_:0.25Dy_2_O_3_	385	(0.412, 0.446)
50ZnO:40B_2_O_3_:5WO_3_:5Nb_2_O_5_:0.5Dy_2_O_3_	385	(0.416, 0.452)
50ZnO:40B_2_O_3_:5WO_3_:5Nb_2_O_5_:0.75Dy_2_O_3_	385	(0.413, 0.448)
50ZnO:40B_2_O_3_:5WO_3_:5Nb_2_O_5_:1Dy_2_O_3_	385	(0.413, 0447)
50ZnO:40B_2_O_3_:5WO_3_:5Nb_2_O_5_:0.5Eu_2_O_3_	394	(0.649, 0.343)
50ZnO:40B_2_O_3_:5WO_3_:5Nb_2_O_5_:0.5Dy_2_O_3_:0.25Eu_2_O_3_	363	(0.442, 0.438)
50ZnO:40B_2_O_3_:5WO_3_:5Nb_2_O_5_:0.5Dy_2_O_3_:0.5Eu_2_O_3_	363	(0.464, 0.424)
50ZnO:40B_2_O_3_:5WO_3_:5Nb_2_O_5_:0.5Dy_2_O_3_:0.75Eu_2_O_3_	363	(0.485, 0.419)
50ZnO:40B_2_O_3_:5WO_3_:5Nb_2_O_5_:0.5Dy_2_O_3_:1Eu_2_O_3_	363	(0.502, 0.408)

## Data Availability

Data are contained within the article.
